# Rheological Behavior of Pectin Gels Obtained from Araçá (*Psidium cattleianum* Sabine) Fruits: Influence of DM, Pectin and Calcium Concentrations

**DOI:** 10.3390/polym14163285

**Published:** 2022-08-12

**Authors:** Sarah da Costa Amaral, Denis Christophe D. Roux, François Caton, Marguerite Rinaudo, Shayla Fernanda Barbieri, Joana Léa Meira Silveira

**Affiliations:** 1 Postgraduate Program in Biochemical Sciences, Sector of Biological Sciences, Federal University of Paraná, Curitiba 81531-980, Brazil; 2 University Grenoble Alpes, CNRS, Grenoble INP, LRP, 38000 Grenoble, France; 3 Biomaterials Applications, 6 Rue Lesdiguières, 38000 Grenoble, France

**Keywords:** Araçá pectin, de-esterification, SEC characterization, calcium gelation, rheology

## Abstract

In this work, purified pectins from Araçá fruits (*Psidium cattleianum* Sabine) were obtained and characterized after partial demethylation. On each prepared sample, the carboxylic yield was obtained by titration, the degree of methylation (DM) by ^1^H-NMR, and the molecular weight distribution by steric exclusion chromatography (SEC). Then, the gelation ability in the presence of calcium counterions was investigated and related to DM (59–0%); the pectin concentration (2–10 g L^−1^); and the CaCl_2_ concentration (0.1–1 mol L^−1^) used for dialysis. The critical pectin concentration for homogeneous gelation was above 2 g L^−1^ when formed against 1 mol L^−1^ CaCl_2_. The elastic modulus (G′) increased with pectin concentration following the relationship G′~C^2.8^ in agreement with rigid physical gel network predictions. The purified samples APP and APP-A with DM ≥ 40% in the same conditions released heterogeneous systems formed of large aggregates. Gels formed against lower concentrations of CaCl_2_ down to 0.1 mol L^−1^ had a higher degree of swelling, indicating electrostatic repulsions between charged chains, thus, counterbalancing the Ca^2+^ cross-linkage. Compression/traction experiments demonstrated that an irreversible change in the gel structure occurred during small compression with an enhancement of the G′ modulus.

## 1. Introduction

The interest of researchers in polysaccharides derived from natural sources has been increasing in accordance with the growing demand for hydrocolloids with potential industrial applications [1,2]. Pectin is one of the most industrially important hydrocolloids, mainly composed of (1→4)-linked-α-D-GalA*p* units, consisting of homogalacturonan (HG) blocks and rhamnogalacturonan type-I (RG-I) blocks, with a backbone made of the repeating [→2)-α-L-Rha*p*-(1→4)-α-D-GalA*p*-(1→]_n_ disaccharide units, which may contain neutral monosaccharide side chains composed predominantly of galactose and arabinose [3,4,5].

The exploitation of pectin gelation for industrial uses is mainly related to a HG rich fraction. The carboxyl groups of the HG in GalA units may be methyl-esterified to different degrees of methylation (DM) and in a pattern which is regulated during plant development [6]. The degree of methyl-esterification influences the gel network formation. Thus, pectins can be classified into (1) high methoxyl pectins (HMP), in which more than 50% of the carboxylic groups of the GalA units are methylated (DM > 50%); (2) low methoxyl pectins (LMP), which present less than 50% of the carboxylic groups methylated (DM < 50%) [3,7,8,9].

The two main gelation mechanisms for pectins are briefly described in the following:

(1) For HMP gelation, a low pH value (pH~3.5) is required to reduce the electrostatic repulsion among pectin molecules due to the decrease in the carboxyl groups of GalA units dissociation, and a high concentration of co-solute (~65% saccharose), which reduces water activity, thereby promoting thermoreversible pectin chain-chain associations based on H-bond stabilization [3,7,10,11,12];
(2) LMP gelation occurs in the presence of bivalent cations, such as Ba^2+^, Sr^2+^, Ca^2+^ but not Mg^2+^. Interaction occurs between Ca^2+^ and two carboxylic sites from antiparallel HG segments that further aggregate laterally to form multimers [7,8]. The junction zones are stabilized through cooperative electrostatic interactions between Ca^2+^ and ionized carboxylic groups of different pectin chains, in agreement with the egg-box model [7,13]. These mechanisms are also described for alginates and other stereoregular carboxylic polysaccharides.

The interaction mechanism of calcium counterions is directly related mainly to the chemical structure of pectins, including the degrees of methyl-esterification and acetylation, and molecular weight [7]. It also depends on the polymer concentration, the distribution of carboxylic groups, and the amount of calcium present in the medium [14]. In addition, the elucidation of the structure–function relationship for gelling properties is challenging because gelation is a complex phenomenon, as electrostatic interactions, entanglement, and cross-linking happen simultaneously [15]. Thus, the study of the pectin–calcium gelling mechanism is still necessary, mainly for polymers obtained from new natural sources that exhibit original chemical structures.

*Psidium cattleianum* Sabine (Araçá) is an edible Brazilian native species of the Myrtaceae family [16,17], which contains several bioactive compounds such as polysaccharides [17,18]. In a previous study, the structural and physicochemical characterization showed that the Araçá pectin (APP sample, i.e., 3.5% *w*/*w* of the initial dried material) is a non-gelling pectin in excess of calcium counterions. Its structure is formed by long homogalacturonan (HG) blocks, partially methylated (initial degree of methyl-esterification DM = 55.9 mol%) and acetylated (2 mol%), with a low proportion of rhamnogalacturonan type I (RG-I) blocks with side chains containing mainly arabinose and galactose. Moreover, after the partial alkaline de-esterification of APP, a strong and homogeneous physical gel is formed, which is stabilized by cooperative GalA^−^-Ca^2+^ junctions for the sample APP-15 (DM = 43.5 mol%), thus, demonstrating an interesting rheological potential for industrial applications in food and/or pharmacy [18]. However, an in-depth study of the rheological properties and gelation mechanism of Araçá pectins is required in order to optimize their applications.

Taking this view, in the present work, the structural and physicochemical characteristics of the de-esterified Araçá pectins were studied in relation to the gelation process. For that purpose, the rheological properties were investigated in order to establish the influence of DM, and the concentrations of pectin and calcium counterions in the formation of the gel network structure of partially de-esterified Araçá pectins.

## 2. Materials and Methods

### 2.1. Materials

Araçá fruits (SisGen nº AE1B38B) were collected and prepared according to Amaral et al. [18]. Briefly, crude pectic fraction from Araçá pulp was obtained by hot water extraction (ACP). Ethanol (99.5%) was purchased from Alphatec. NaN_3_ (>99%); ethanol (P.A., ≥99.9%); and HCl 1 mol L^−1^ (1 N) Titripur^®^ were purchased from Merck (Darmstadt, Germany). NaCl (≥99%); NaOH (≥97%); and NaNO_2_ (≥99%) were purchased from Sigma-Aldrich (St. Louis, MO, USA). Slide-A-Lyzer Dialysis cassette MWCO 2000 D cut-off membrane was acquired from Thermo Scientific (Rockford, IL, USA).

### 2.2. Purification and De-Esterification of Pectin

**Figure 1:**
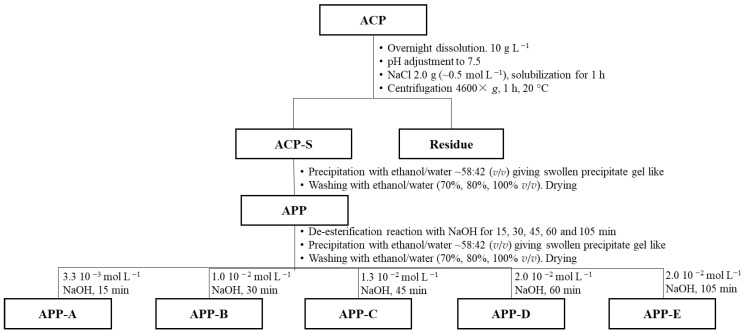
Scheme of purification and de-esterification of pectins from the pulp of Araçá (*Psidium cattleianum* Sabine) fruits.

The ACP (Araçá crude pectin) was purified according to Amaral et al. [18] and Rinaudo [19] with some improvements (Figure 1): 1 g of ACP was solubilized overnight (16 h) in 100 mL of deionized water under magnetic stirring. Then, the pH was adjusted to 7.5 with a 0.1 mol L^−1^ NaOH solution and 2.0 g of NaCl was added (~0.5 mol L^−1^) under stirring. The solution was left to stir for 1 h to obtain the sodium form of charged polymers, followed by centrifugation (4600× *g*, 1 h, at 20 °C) to remove water-insoluble impurities, thus, obtaining ACP-S. After centrifugation, the Araçá purified pectin (APP) was obtained by ethanol precipitation at a concentration of around 60% (ethanol/water *v*/*v*), isolation of the precipitate, and dispersion in increasing concentrations of ethanol/water mixtures (70%, 80% *v*/*v*) and, at the end, with ethanol P.A. for the deswelling step. Finally, the samples were dried at atmospheric conditions (~1 atm, 25 °C) until a constant weight was achieved (Figure 1).

The samples with different degrees of methyl-esterification (DM) were prepared by de-esterification reaction with different times and concentrations of NaOH, as described in Figure 1, for 15 min (APP-A); 30 min (APP-B); 45 min (APP-C); 60 min (APP-D); and 105 min (APP-E). The reaction was halted by 0.1 mol L^−1^ HCl until reaching pH 7.5. Then, ethanol precipitation and drying of APP de-esterified samples were carried out, as described for APP (Figure 1).

### 2.3. Uronic Acid Content and Degree of Methyl-Esterification (DM)

The uronic acid content and the degree of methyl-esterification (DM) were quantified by the conductometric titration technique described previously [9,18,20], with a conductometer Thermo Scientific, Orion Star A215. As described previously [18], the value of methyl-esterified uronic acid (GalA-Met) was determined by the difference between the uronic acid content after total de-esterification (total GalA) and the initial carboxylic groups content (GalA^−^Na^+^). The degree of methyl-esterification (DM) was calculated through the ratio between GalA-Met and the total GalA expressed in molarity. The values obtained from g/g or mol/mol were normalized considering the molar mass of 198 g mol^−1^, 190 g mol^−1^, and 162 g mol^−1^ for GalA^−^Na^+^, GalA-Met, and neutral monosaccharides, respectively, and multiplied by 100.

### 2.4. Size Exclusion Chromatography

Samples were dissolved in water (4 mg mL^−1^) or in 0.1 mol L^−1^ NaNO_3_ under stirring for 24 h and filtrated on a 0.2 µm porous membrane before injection. The weight-average molecular weights (M*_w_*) were determined through size exclusion chromatography (SEC) measurements using two columns in a series from Shodex SB 806 M HQ, associated with a pre-column SB-G 6B. A laser light scattering miniDAWN TREOS detector (Wyatt Technology Corporation, Santa Barbara, CA, USA) was associated with a Wyatt differential refractometer. The eluent was made of 0.1 mol L^−1^ NaNO_3_ with 0.3 g L^−1^ NaN_3_ with a flow rate of 0.5 mL min^−1^ at 30 °C. The dn/dc was equal to 0.155.

### 2.5. Nuclear Magnetic Resonance (NMR) Spectroscopy

Mono-dimensional ^1^H-NMR measurements were performed at 353 °K on a Bruker AVANCE I instrument equipped with a 5-mm probe operating at 400.2 MHz (Bruker, Billerica, MA, USA). A total of 3 mg of polymer was dissolved in 0.5 mL of D_2_O, and 32 scans were recorded. Chemical shifts of the protons were expressed as δ (ppm) using H_2_O at 4.25 ppm at 80 °C as reference. The data were collected and processed using the Software TOPSPIN, version 3.1 (Bruker Biospin, Rheinstetten, Germany).

The degree of methyl-esterification (DM) values was determined by ^1^H-NMR spectroscopy, integrating the hydrogen areas corresponding to -CH_3_ at 3.9 ppm, H-1 (5.1–5.3 ppm), H-5 of unesterified α-D-GalA*p* units at 4.7 ppm, and H-5′ of esterified α-D-GalA*p* units at 5 ppm, as discussed by Grasdalen, Bakøy, and Larsen [21]. The hydrogen from -CH_3_ around 3.9 ppm gives DM, using H-1 integral as reference [22]. Nevertheless, the H-1 signal of Gal-A units is very near the H-1 of arabinogalactan. The DM % is also determined by the ratio of -CH_3_ using (H-5 + H-5′) integrals or from the ratio H-5′/(H-5 + H-5′) with a relatively good accordance.

### 2.6. Rheological Characterization

Araçá pectin gels were prepared using different conditions: samples with different DM (APP, APP-A, APP-B, and APP-E) at 10 g L^−1^, and APP-E at different concentrations (2, 3.3, 6.7, and 10 g L^−1^) were previously dissolved for 17 h in deionized water, and 3 mL of each solution was placed into a dialysis cassette (Slide-A-Lyzer, MWCO 2000 D from Thermo Scientific^TM^, Norcross, GA, USA) maintained vertically and dialyzed against 150 mL of 1 mol L^−1^ CaCl_2_ solution for 24 h [18,23]. APP-E at 10 g L^−1^ was previously dissolved for 17 h in deionized water, and 3 mL of the sample was placed in dialysis against different concentrations of CaCl_2_ (0.1, 0.5, 1.0 mol L^−1^) solutions for 24 h. The resulting formed gels were recovered by cutting off the dialysis membrane with a scalpel. The gel formed was weighted and then punched into 10-mm diameter discs and cut down to 2–3 mm thickness before being immersed into a CaCl_2_ solution for stabilization before the rheological measurements.

Rheological characterizations of the gels were performed on an ARES-G2 rheometer from TA Instruments Company. The APP-B, APP-D, and APP-E gels were loaded between the two rough plates separated by a thickness-matched gap adapted to a 10-mm diameter. To avoid evaporation and consequent change in concentration, the same CaCl_2_ solution used in the dialysis of each sample was used to immerse the gels during the rheometer analysis. The immersion was made possible by the addition of a large diameter glass cylinder positioned concentrically to the rheometer tool and sealed by silicone grease positioned at its base. All the experiments were performed at 25 °C using the Advanced Peltier System (APS) controlled environmental system. The APP, APP-A, and ACP solutions were analyzed on a MCR 501 rheometer from Anton-Paar equipped with a geometry, consisting of two 50-mm diameter smooth titanium plates at a 1-mm gap in order to determine the viscosity.

**Figure 2:**
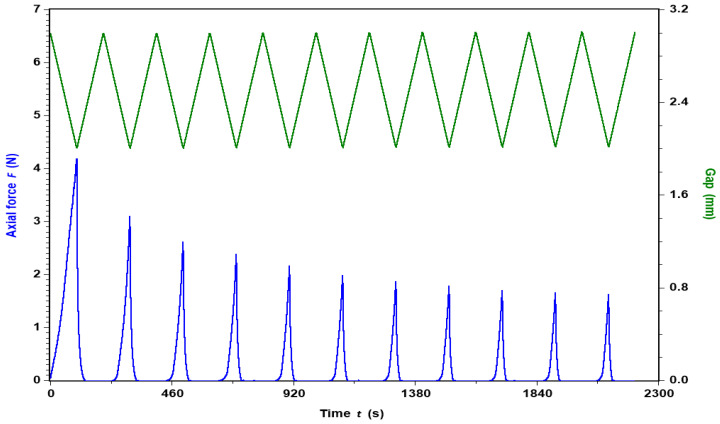
Fatigue test applied onto APP-E at pectin concentration of 10 g L^−1^ in 1 mol L^−1^ CaCl_2_ and at T = 25 °C.

In addition to the usual flow and the dynamic rheology, an original study of successive compression/tension on gels was performed on an ARES-G2 rheometer from TA Instruments Company, equipped with the same 10 mm diameter rough tools. Initially, each gel was loaded in order to ensure contact between the plates and the gel. The contact was checked visually, as well as by the presence of axial force which stabilized in a few minutes around 0.04 N. The test consisted of repeating successive downward and upward gap changes at 0.01 mm/s over a displacement of 1 mm, as shown in Figure 2. Mechanically, this corresponds to a fatigue test. During those ramps in time, the axial force was recorded. For all the gels tested, the axial force increased for a downward gap change and decreased for an upward gap change. For all the gels tested, a decrease over time in the value of the maximal axial force obtained at the minimum gap was observed. Over time, this value decreased for all the studied gels, reaching a constant value equal to 1.5 N, as shown in Figure 2.

At t = 0 s, the axial force is about 0.04 N for an initial gap of 3 mm before reaching 4.2 N at 3 mm by applying a gap change of 0.01 mm/s. When the gap returns to its initial value of 3 mm, the axial force does not return to its initial value but pulls the top plate down with an axial force of about −0.04 N. Over time, the negative value of the axial force at the upper gap position will be present, but this is not be investigated in this study, in part because of its small change in magnitude.

### 2.7. Effective Concentration and Swelling Degree

After dialysis of 3 mL containing a known amount of pectins in the dialysis cell, the total gel was weighted. In all cases, a contraction was observed for the transition sol-gel. From the swollen weight and the initial dried weight, the effective concentration was determined from the volume of the solution included in the gel and the dried weight of material. The degree of swelling was expressed in mL of solution per gram of dried material.

## 3. Results and Discussion

### 3.1. Structural Characterization of the Pectins from Araçá Pulp

In order to obtain a purified pectin from the Araçá crude fraction, the ACP fraction was purified as described before [18] with some modifications (Figure 1) to obtain the APP sample under the sodium form. This method was found to provide pure pectins, excluding the presence of a neutral polysaccharide after analysis by DEAE chromatography. Then, in order to obtain samples with different degrees of methyl-esterification (DM), the APP sample was de-esterified with different concentrations of NaOH and during different times (Figure 1).

The values of GalA and DM obtained by conductometric titration are summarized in Table 1. The value obtained for total GalA increased slightly (up to 87% and following 13% of neutral sugars) when compared to the total GalA values (71.9%) reported before [18]. This might be explained by the increase in NaCl concentration used in this work (0.5 mol L^−1^ instead of 0.08 mol L^−1^ used previously) in the purification step, which may have removed more counterions interacting more strongly with carboxyl groups (multivalent counterions). The DM value (59%) for the purified sample, referred to as APP, was also slightly higher than previously reported (55.9%) for the purified sample, also referred to as APP [18]. On the other hand, the yield of APP recovered from ACP using a larger concentration of NaCl (Figure 1) decreased from 75% to 69% (*w*/*w*), probably due to a better exchange of counterions (for Na form), thus, avoiding precipitation of a calcium salt form. It also justifies the larger value of DM of this purified APP.

The obtained purified sample was then classified as a high methoxyl (HM) type [7,24]. It was demonstrated in this work that it does not form gels in calcium excess. Therefore, to carry out the study of the interaction with calcium, pectins with different DM were produced from APP. The partial de-esterification reaction using different reaction times and NaOH concentrations (Figure 1) produced the samples APP-A, APP-B, APP-C, APP-D, and APP-E, as reported in Table 1.

### 3.2. NMR Structure Characterization

Following the analysis performed in our previous work [18], and considering the analysis proposed by Grasdalen et al. [21] and Winning et al. [25], the different samples prepared were characterized by ^1^H-NMR. The main results are given in Table 2.

The results show that there is not a good agreement between the titration and the NMR analysis. Indeed, the interpretation of titration is complex in some conditions. Likewise, the NMR signal of -CH_3_ overlaps with other structural protons, and it is difficult to quantify it precisely. This is the reason why a small amount of methyl groups were identified by NMR (Table 2) and not by titration. As shown before, the low acetyl content decreases simultaneously with methyl groups in the presence of alkaline medium. From the high ratio GalA/Rha, it was confirmed that those pectins contain large blocks of GalA unit (HG) interrupted by a few GalA-Rhamnose blocks (RG-I). Neutral carbohydrates, including arabinogalactan and rhamnose units, represent around 22% of the total GalA (mol/mol), thus, allowing the deduction that neutral carbohydrates represent 18 g/100 g of the total GalA, which means around 16 g in 100 g of initial pectin. This content agrees with the result obtained by titration based on 87% (*w*/*w*) of the total GalA.

### 3.3. Molecular Weight Characterization

#### 3.3.1. Solubilization in 0.1 mol L^−1^ NaNO_3_

**Figure 3:**
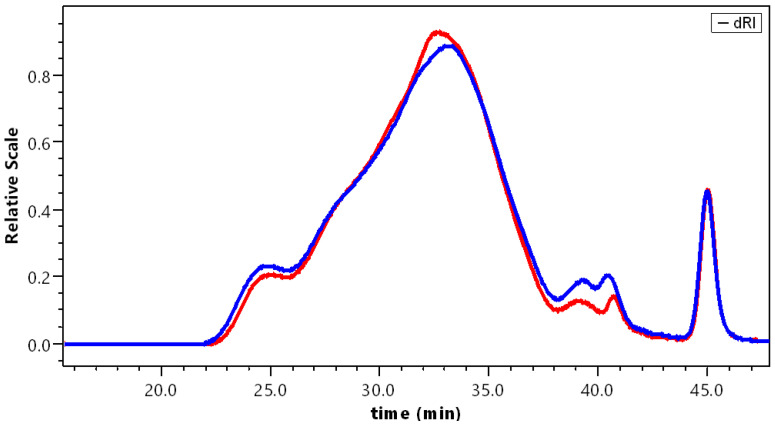
Reproducibility test: SEC chromatograms for two APP preparations dissolved in 0.1 mol L^−1^ NaNO_3_.

In Figure 3, the chromatograms obtained from two independent purifications of the ACP allowed us to show the good reproducibility of the adopted preparation processes (Figure 1). It indicates the presence of high molecular weight aggregates (around 2 ± 0.1%). The large peak presents a shoulder by a 28 min elution, indicating the presence of two polymer families, being roughly determined.

**Figure 4:**
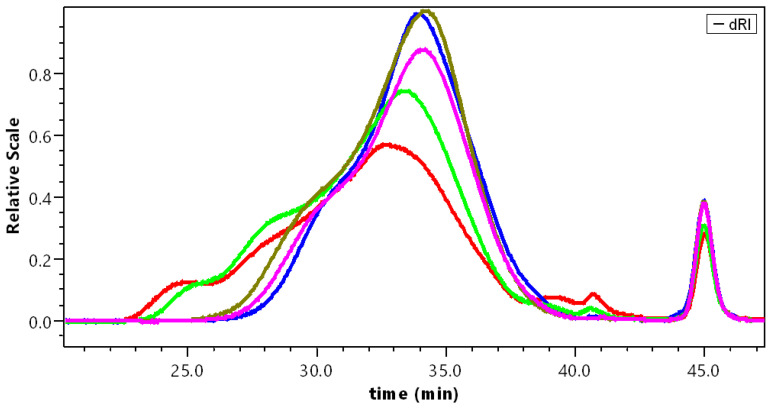
Chromatograms of the different pectins dissolved in 0.1 mol L^−1^ NaNO_3_. APP (red); APP-A (green); APP-B (brown); APP-C (pink); and APP-E (blue).

The different pectins were studied in the same conditions. Their chromatograms are given in Figure 4. In this figure, it is clear that a strong evolution of the samples occurs as soon as demethylation starts. APP-A was still polydispersed and a small fraction of aggregates remained. Then, when demethylation proceeded, APP-B to APP-E nearly overlapped, as found in our previous work [18]. The analysis of the data is given in Table 3.

#### 3.3.2. Solubilization in Water

**Figure 5:**
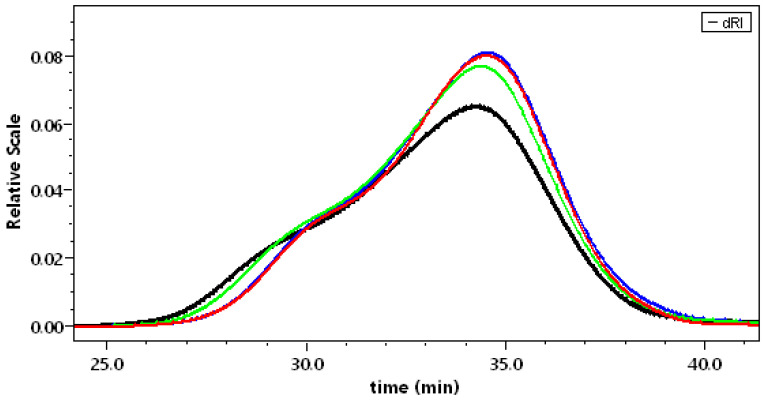
Chromatograms of pectins with different DM solubilized in water. APP-B (black); APP-C (green); APP-D (red); and APP-E (blue).

The same samples were dissolved in water to increase their solubility due to the increase in electrostatic repulsion for pectins in the sodium salt form. In Figure 5, the chromatograms obtained for APP-B to APP-E confirm the overlapping of the curves when demethylation increases, as found previously [18], and after dissolution in 0.1 mol L^−1^ NaNO_3_.

In summary, the results obtained after solubilization in the presence of salt or in water are compared in Table 3. When DM decreases and for DM < 30%, the molecular weight distributions overlap in the two solvents tested, allowing the identification of two series of polymers for which the two fractions are roughly estimated. (1) When solubilized in water, a fraction of around 15 w% of the samples was eluted first with a high M*_w_* (2 × 10^6^ g mol^−1^), and the second fraction, 85 w%, was eluted from APP-B to APP-E with M*_w_* = 188,000, 150,000, 135,000 and 142,000 g mol^−1^, respectively. (2) When solubilized in 0.1 mol L^−1^ NaNO_3_, the first fraction, around 20 w%, was eluted with a high M*_w_* (2.5 × 10^6^ g mol^−1^), and the second one, around 80 w%, for APP-B, APP-C, and APP-E with M*_w_* = 183,000, 174,000, 156,000 g mol^−1^. The M*_w_* values are almost identical in the two solvents tested. These results are in accordance with our previous data [18]. They indicate that there is still some aggregation in the APP sample even after filtration through 0.2 µm porous membrane, as discussed previously [26].

As soon as the pectins were in contact with NaOH, they dissolved better, thus, suppressing the high M*_w_* fraction (larger than 2 × 10^6^) (Figure 3 and Figure 4). After longer contact with NaOH, the M*_w_* did not clearly decrease, meaning that depolymerization did not occur. The only difference between the two sets of experiments was that the signal corresponding to the larger weight fraction of chains was slightly larger for dissolution in water.

For comparison, it is shown that dissolution of the different samples is significant once the products are properly purified. The aggregates in the different samples are better dissociated in water, as indicated by lower M*_w_*, especially for APP and APP-A, in which a small amount of high M*_w_* exists. Then, the weight-average molecular weight M*_w_* is lower after dissolution in water, with partial disappearance of the first small fraction eluted between 24 and 25 min, present in NaNO_3_ (Figure 4). Regarding the fraction of material eluted after filtration on 0.2 µm porous membrane, it remains in the same order and increases for APP-C to APP-E up to 80 w%, which is a good result for SEC pectin in relation to the proposed purification process.

### 3.4. Rheological Characterization of Araçá Pectins

As the potential of Araçá pectins for gel formation was demonstrated in the previous work [18], in the next part of this work, a more in-depth study on the rheological characterization of those original pectins was carried out to investigate the influence of DM, pectin concentration, and calcium ion concentration on the gel characteristics.

**Figure 6:**
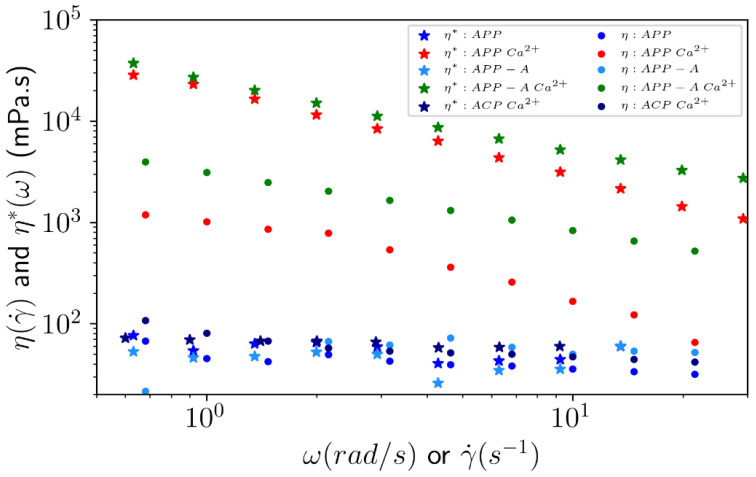
Shear (dots) and dynamic (stars) viscosity as a function of the shear rate and angular frequency, respectively, for ACP, APP, and APP-A, prepared under sodium salt form at 10 g L^−1^, with or without dialysis against 1 mol L^−1^ CaCl_2_ at 25 °C.

Initially, the flow behavior was evaluated through the viscosity curves as a function of the shear rate for the APP (HM pectin) and the APP-A (LM pectin) samples under a Na salt form, with or without dialysis, against 1 mol L^−1^ CaCl_2_ (Figure 6).

Their behavior was compared with that of the ACP sample. Even in excess of Ca^2+^, ACP had almost the same viscosity as APP and APP-A under their sodium salt form, with no dependence on the shear rate (Newtonian behavior). A shear thinning behavior was observed for APP (red markers, DM = 59%) and APP-A (green markers, DM = 36.8%) after dialysis against 1 mol L^−1^ CaCl_2_. In addition, the viscosities were higher compared to the sodium salt form and decreased when the shear rate increased (shear thinning behavior), especially for APP-A with a partial demethylation. The non-linear dependence in γ observed with APP and APP-A was in fact related to the heterogeneity of this system containing macro-aggregates, observed under microscope in excess of calcium. This suggests that only a few blocks of free carboxylic units along pectin chains exist in APP and APP-A, thus, avoiding the stabilization of an infinite network in the presence of CaCl_2,_ even in large excess.

**Figure 7:**
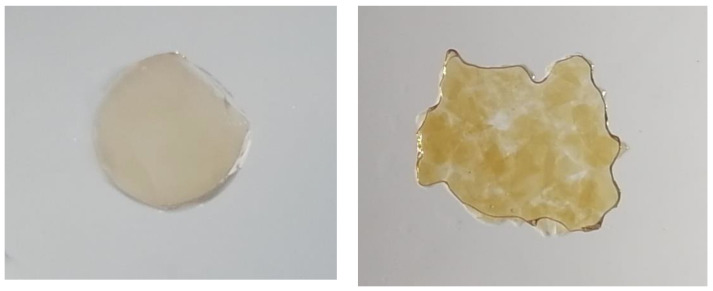
Examples of homogeneous gel (**left** image with a DM~0, APP-E) and inhomogeneous gel (**right** image with a DM~59%, APP-Ca^2+^). Sizes of the samples were around 10 mm in diameter.

In the absence of calcium, the viscosity values at 1 s ^−1^ were 38 ± 2 mPa and 56 ± 2 mPa for APP and APP-A, respectively, and were nearly equal to the ACP sample viscosity obtained before the purification process in the presence of calcium (Figure 6). This last result indicates that there were no free carboxylic blocks along the chains corresponding to higher DM and/or the presence of multivalent counterions. In fact, no gelation was observed on ACP and only large aggregates were formed on the APP and APP-A samples, as shown in Figure 7.

Dynamic viscosities (η*) were carried out on the same samples, confirming that even in excess of CaCl_2_, ACP had almost the same viscosity as APP and APP-A in their sodium salt form, with no dependence on the shear rate (Newtonian behavior). For APP and APP-A, the dynamic viscosities were higher than for ACP, showing shear thinning behavior. The non-overlap of the shear and dynamic viscosities of APP and APP-A in the presence of CaCl_2_ (Figure 6) indicates that the samples were not homogeneous, as shown in Figure 7.

**Figure 8:**
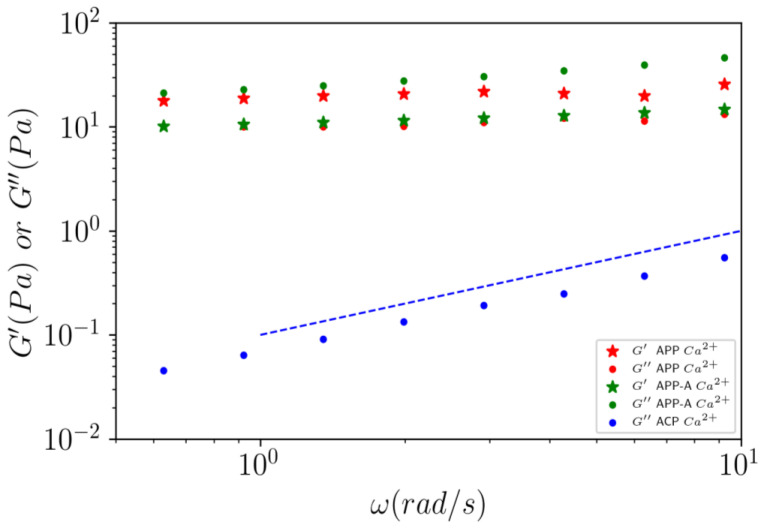
Elastic (G′) and viscous (G″) moduli as a function of the angular frequency (ω) for ACP, APP, and APP-A at 10 g L^−1^, against CaCl_2_ at 25 °C for an applied oscillatory strain γ = 0.1%. Full dots correspond to viscous modulus G″ (Pa) and stars to the elastic modulus G′ (Pa). No elastic modulus is shown for ACP because it behaves like a Newtonian liquid. Dashed line has a slope as a Newtonian liquid should have for dynamic measurements at a low oscillatory strain.

The dynamic measurements were also able to characterize the viscoelastic behavior of ACP, APP, and APP-A after dialysis against 1 mol L^−1^ CaCl_2_ (Figure 8).

For the samples APP and APP-A, in the absence of CaCl_2_ excess, viscous G″ moduli were in the same range of values (data not shown) as ACP and followed a linear behavior in angular frequency, indicating a Newtonian behavior and confirming the results observed in Figure 6.

In excess of calcium, the moduli increased greatly to around 10 Pa in comparison to the samples without calcium (Figure 8). Furthermore, the moduli were almost independent of frequency, indicating that a loose gel forms in the APP and APP-A samples under a small strain. For APP-A in the presence of calcium, the viscous modulus was slightly higher than the elastic one, which can be explained by the formation of an incomplete gel. Similarly, the viscous modulus G″ of APP-A was slightly higher than that of APP, due to the higher partial demethylation and higher yield in -COO^−^Na^+^ groups.

In conclusion, for 10 g L^−1^ pectin solutions against 0.1 mol L^−1^ CaCl_2_, (i) the initial ACP pectin is a non-gelling pectin in calcium excess; (ii) after our purification process with DM = 59% (APP), and after partial demethylation with DM = 36.8% (APP-A), a heterogeneous dispersion made of large aggregates was formed in excess of calcium, and it was observed with G′ < G″ almost independent of the frequency, except for APP; (iii) in the presence of Ca^2+^, the shear viscosity η and the dynamic viscosity η* were not superimposed (η* > η), indicating a heterogeneous system, as confirmed by microscope.

#### 3.4.1. Effect of DM on Gels

**Figure 9:**
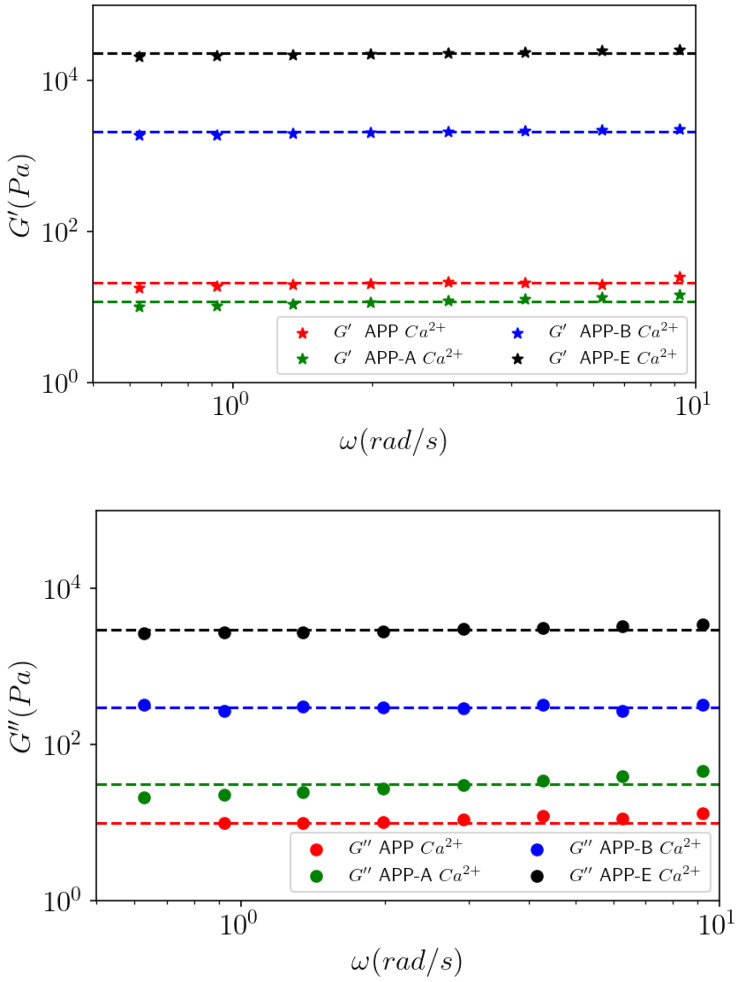
Effect of DM on the viscoelastic behavior. Frequency sweep of APP, APP-A, APP-B, and APP-E at 10 g L^−1^ in 1 mol L^−1^ CaCl_2_ at 25 °C for an applied oscillatory strain γ = 0.1%. Stars correspond to the elastic modulus G′ (Pa) and full dots correspond to the viscous modulus G″ (Pa).

According to the literature [3,7], the degree of methyl-esterification (DM) of pectins influences the formation of a gel network. Therefore, the effect of DM on the viscoelastic behavior of the HM pectin (APP) and the de-esterified APP-A, APP-B, and APP-E samples in the presence of CaCl_2_, was examined in Figure 9. The role of DM was made possible because the molecular weights are almost the same on all the samples, as shown by SEC in the sample during the progressive de-esterification.

The partly de-esterified sample APP-A sample showed that G″ was higher than G′ over the analyzed frequency in excess of calcium, which is a characteristic of a liquid-like behavior. This sample did not form a homogeneous gel either, although large aggregates could be observed, indicating that parts of the sample structure formed interactions against the calcium solution. In fact, this means that there are only a few blocks of demethylated galacturonic units, thus, allowing loose interchain interactions depending on the polysaccharide concentration.

In contrast, the APP-B and APP-E samples showed a typical gel behavior in which the G′ modulus remained higher than the G″ modulus, which was almost parallel and constant throughout the analyzed frequency range. In addition, the G′ values increased sharply and showed an increase in the effective concentration, as expressed in Table 4. The values of such a contraction are not available for APP and APP-A because they did not form a continuous gel, thus, resulting in random results.

**Figure 10:**
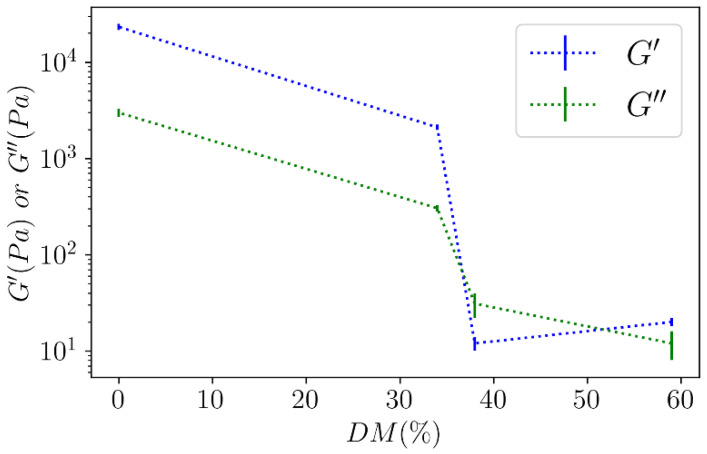
The effect of DM on the viscoelastic behavior on values of G′ and G″ as a function of DM of Araçá pectins.

From the G′ and G″ moduli at different degrees of methylation after the dialysis of 10 g L^−1^ solutions against in 1 mol L^−1^ CaCl_2_ shown in Figure 9, Figure 10 shows a strong transition of around DM = 35% with a large increase in the moduli in a short range of DM.

From DM = 40% to 30%, G′ passes from 10^1^ to 10^4^ Pa, thus, showing that the formation of the gel network is favored in this DM range. At the same time, a large contraction of the gel is observed (Table 4). Such a transition was previously observed by Ralet et al. [27] for citrus pectin with a random distribution of free galacturonic acids able to dimerize below DM~35%. Fraeye et al. [28] also demonstrated for apple pectin that rheological characteristics depend strongly on DM. A transition of moduli was observed from DM = 46% to 31%. A transition was also obtained on pectins with different degrees of esterification using the activity coefficient of cadmium [29] or calcium [30,31].

Strong gels form when the number of the demethylated galacturonic blocks increases, as well as their length, as demonstrated by Kohn [32] and Ngouémazong et al. [33]. It has been discussed that 4 to 5 cooperative calcium zones must be formed to have stable cross-linking in these physical gels [14].

#### 3.4.2. Effect of Pectin Concentration on Gelation

**Figure 11:**
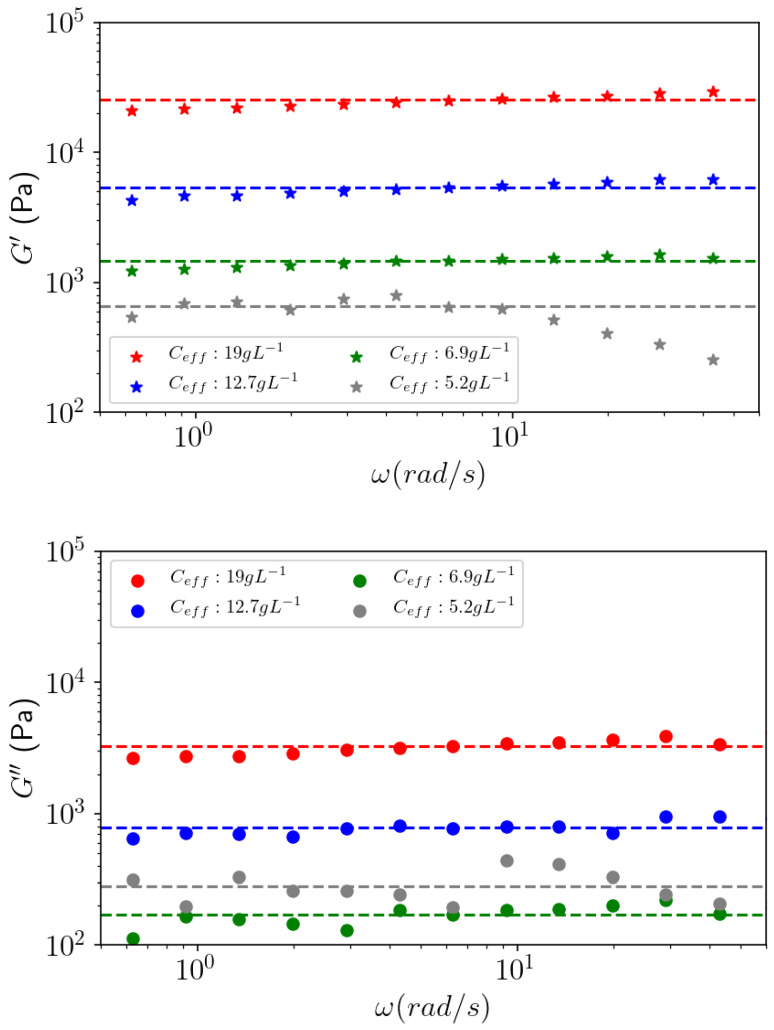
Influence of the effective APP-E pectin effective concentration on the viscoelastic behavior (G′ and G″) of gels as a function of the frequency for an applied oscillatory strain γ = 0.1%.

The gelation of pectins also depends on the polysaccharide concentration at a given DM and fixed calcium concentration. It occurs over a critical polymer concentration, mentioned previously for Araçá, around 2 g L^−1^ [18]. In the following, the role of Araçá pectin concentration in calcium gelation in 1 mol L^−1^ CaCl_2_ was studied. For that purpose, the de-esterified APP-E sample was chosen to produce gels at different prepared concentrations (2–10 g L^−1^). At all the tested concentrations, a typical gel behavior can be observed (Figure 11), with G′ higher than G″ over the frequency analyzed range, except for 2 g L^−1^ solution (or effective concentration 5.2 g L^−1^), which more likely behaves like a viscoelastic fluid. As the pectin concentration increases, the gel swelling decreases, indicating a contraction of these gels for which an effective concentration was recalculated (Table 5).

**Figure 12:**
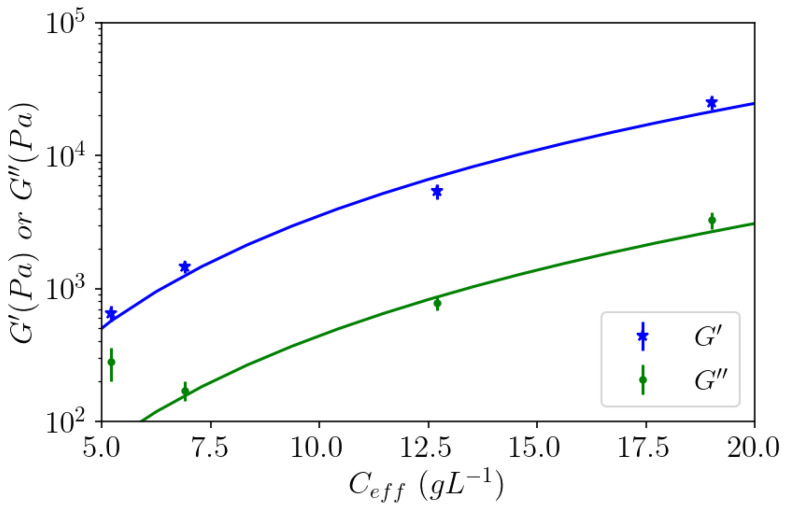
Elastic and viscous moduli for APP-E as a function of the effective concentration obtained at low angular frequencies. (Values correspond to the dashed lines in Figure 11). Continuous lines correspond to a power law fit~C_eff_^2.8^.

From these data, it can be observed that stronger gels are formed as the concentration of APP-E increases (Figure 11). At 10, 6.7, and 3.3 g L^−1^ of the prepared concentration, G′ was approximately 10 times higher than G″. In the observed frequency range, those moduli are constant for each concentration and increase with an increasing pectin concentration such as G′~C_eff_^2.8^ (Figure 12). The exponent 2.8 remains in the same order of magnitude of 2.26 if we take the concentration instead of the effective concentration.

This dependence in concentration can be compared with the G′ dependence in polymer concentration obtained from different physical gels consisting of stiff junction zones between them, with an exponent of around 2 for κ-carageenans in 0.1 mol L^−1^ KCl [34] and for alginate in excess of CaCl_2_ [35]. Exponents 3 and 2.16 were also obtained for a polyacrylamide and agarose gels, respectively [36,37]. These results must be compared to theoretical predictions of the elastic modulus as a function of the concentration, taking into account the rigidity of the polymer, the number of cross-links, and the rigidity of the cross-links. Indeed, the deformation of a network made of rigid elements shows an evolution of the modulus as a function of the concentration evolving, with a power 2 for rod-like chains and a power 5 for a “frozen random walk polymer” [38].

**Figure 13:**
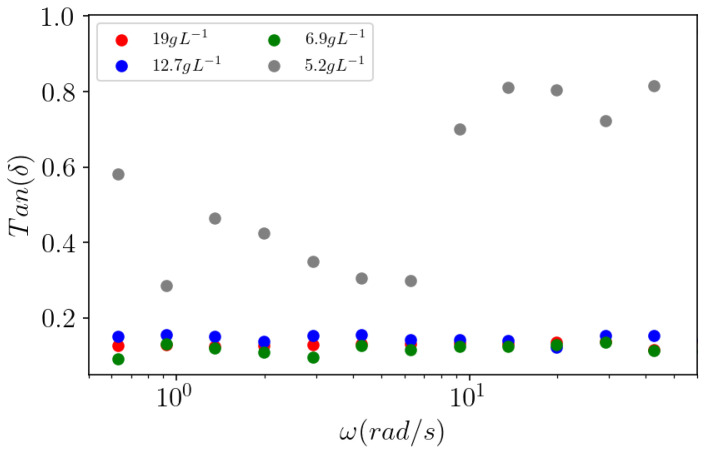
Tan δ = G″/G′ as a function of the pulsation at applied strain γ = 0.1 for APP-E pectins with effective concentrations from 5.2 g L^−1^ to 19 g L^−1^.

In the literature, viscoelastic behavior is often characterized by Tan δ. In Figure 13, Tan δ is plotted as a function of angular frequency based on the data of Figure 11. Over the 2 g L^−1^ solution, Tan δ~0.1 is low, which corresponds to strong gels and a large contraction (or low swelling), as indicated in Table 5.

In Figure 11, considering the solution prepared at 2 g L^−1^ (5.2 g L^−1^ in effective concentration), the behavior looks like a loose gel with irregular moduli dependence with frequency. This is most clearly seen in Figure 13 on Tan δ = G″/G′ which is irregular with the angular frequency, thus, confirming the heterogeneity of the gel obtained at this concentration. At a higher effective concentration, Tan δ ≈ 0.1 is in accordance with the behavior of a strong gel.

From these results, the critical polymer concentration to obtain strong gels in excess of calcium is over 2 g L^−1^ as prepared (5.9 g L^−1^ in effective concentration), which confirms previous data [18].

From strong gels at a high concentration, the gel is homogeneous, allowing the use of G′ to approximate the porosity of the gel formed, considering the following relation:

(1) $$G_{0}=C\;R\;T\;\frac{C\;R\;T}{M_{e}}$$

in which *G*_0_ is taken as the G′ value in the elastic plateau, *C* is the polymer concentration, *R* the Boltzmann constant, *T* the absolute temperature, and *M_e_* is the molar mass between two entanglements or cross-link points (related to a length for a known chemical structure).

When applying this formula for the higher concentration, it results in a pore size of 10 nm; the pore size increases as the effective concentration decreases, as expected. Moreover, its size is of the same order of magnitude as the pores obtained with alginates in excess of calcium by inverse steric chromatography [39], where beads with pores from 11.2 to 20 nm, measured after drying, were obtained by dropping alginate solutions into excess of CaCl_2_ solutions.

In conclusion, under demethylated form, it is shown that: (i) a strong homogeneous gel was formed after dialysis against 1 mol L^−1^ CaCl_2_ as soon as DM < 40%; (ii) a critical Araçá pectin initial concentration C* > 2 g L^−1^ was necessary in order to obtain a continuous gel associated with a strong contraction, compared with the initial concentration prepared; (iii) the elastic modulus of the gel obtained varied as C_eff_^2.8^, in good accordance with previous results obtained from physical gels and in accordance with theoretical predictions.

#### 3.4.3. Effect of Calcium Concentration on Gels

**Figure 14:**
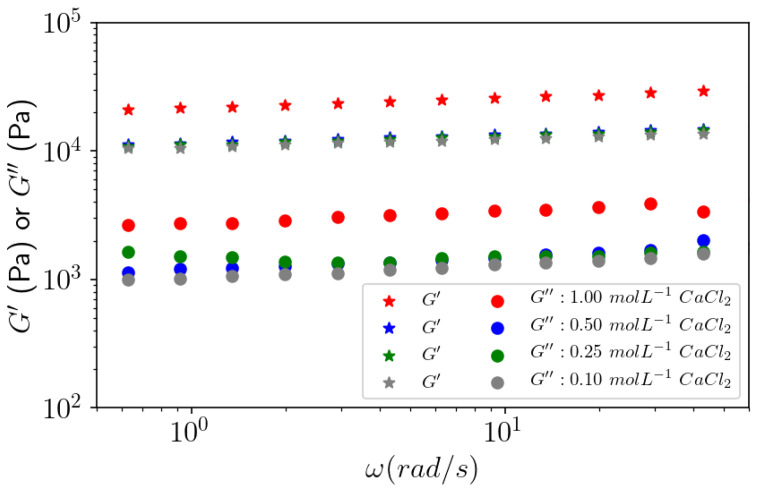
Effect of calcium ion concentration on the viscoelastic behavior of G′ and G″ as a function of the frequency sweep. APP-E at 10 g L^−1^ dialyzed against different concentrations of CaCl_2_ at 25 °C for an applied oscillatory strain γ = 0.1%.

In contrast to what we observed for the change in the pectin concentration, which drastically influenced the gel strength, the change in the calcium concentration did not show a great impact on the G′ and G″ moduli throughout the measured frequency range (Figure 14), except for 1 mol L^−1^ CaCl_2_. These results agree with the contraction given in Table 6, which shows the degree of swelling as a function of CaCl_2_ concentration showing a lower degree of swelling for 1 mol L^−1^ CaCl_2_.

These results indicate that gels formed against CaCl_2_ with a lower concentration down to 0.1 mol L^−1^ (even in a large excess, compared to the carboxylic content in the sample able to associate with calcium) have a higher degree of swelling, indicating electrostatic repulsions between charged chains counteracting the Ca^2+^ cross-linking. As the ionic concentration increases, those long-range electrostatic repulsions decrease at the same time as the degree of swelling.

Regarding the gelation process and, in particular, the homogeneity and spatial distribution of the gel formed, Bouffar-Roupe [35] showed on a dialysis of alginate in 1 mol L^−1^ CaCl_2_ in a cellulosic cylinder bag that a cylindrical gel is formed, with higher concentrations of CaCl_2_ and polymer at the periphery than in the center of the cylinder. Such distribution was avoided with the planar dialysis cells, thus, allowing a large exchange area to obtain a better homogeneity of the gel in the present work.

#### 3.4.4. Compression

**Figure 15:**
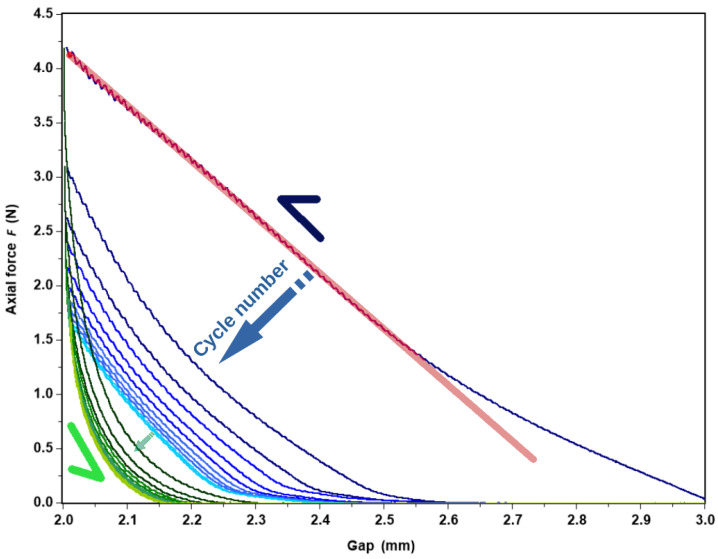
Axial force as a function of gap under a fatigue stress condition, successive compression (blue curves)/traction (green curves) for APP-E gel formed at 10 g L^−1^ and dialyzed against 1 mol L^−1^ CaCl_2_. The half arrows indicate the directions of the cycles and the full arrows the evolution of the axial force as a function of the gap as a new compression/traction cycle is applied. The red line corresponds to the best fit obtained on the compression curve at low gap values, thus, allowing us to extract the Young modulus E (see text).

An original test to characterize the gel is proposed by a successive time cycle of compression/tension (Figure 2). Figure 15 shows the axial force as a function of the applied gap for 11 compression/traction cycles onto the APP-E gel formed at 10 g L^−1^ and dialyzed against 1 mol L^−1^ CaCl_2_. During the traction/compression test, the gel remained immersed in excess of selected solvent during the experiment. In Figure 15, the first compression curve (in blue) is different than the following compression curves (also in blue but clearer).

The analysis started at an almost 3-mm gap with an axial force of less than 0.1 N, in order to reach a 2-mm gap with an axial force of around 4.25 N. From there, the stress was computed as the ratio between the force and the surface of the plate: σ = F/S, and the strain was calculated as the ratio between the change of the gap and the initial gap: ε = (g – g_0_)/g_0_, where g is the actual gap and g_0_ is the initial gap. By definition, the E-modulus is then equal to the ratio between the stress and the strain: E = σ/ε.

**Figure 16:**
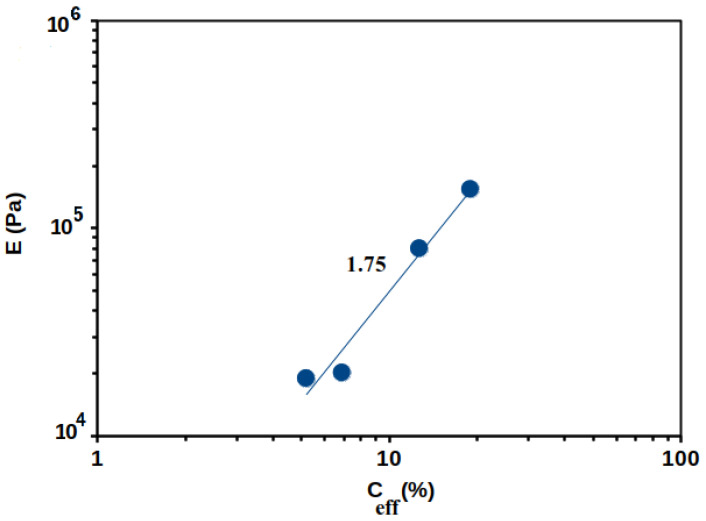
Elastic modulus dependence in effective concentration obtained from the slope of the red line at low compression gap (cf. Figure 15) for APP-E against 1 mol L^−1^ CaCl_2_. Continuous blue line corresponds to the power law fit following an exponent 1.75.

In Figure 16, the E-modulus is plotted as a function of the effective concentration for all the first compression curves. It follows a power law E~C^1.75^ behavior close to the one obtained by Normand et al. [37], who obtained E~C^1.7^ for an agarose gel in compression.

However, if we refer to Figure 15, we can note different points. With each cycle, the hysteresis corresponding to the area above the compression and the traction curve decreases. The decrease in the hysteresis is mainly a consequence of the plastic deformation of the sample leading to a decrease in the gap value, which is given by the gap value at zero axial force; for example, the gap is equal to ~2.6 mm at the beginning of the second cycle. This repeating evolution, known as cyclic fatigue damage, may refer to « degradation » properties of the sample [40,41], attributed to a structural change.

**Figure 17:**
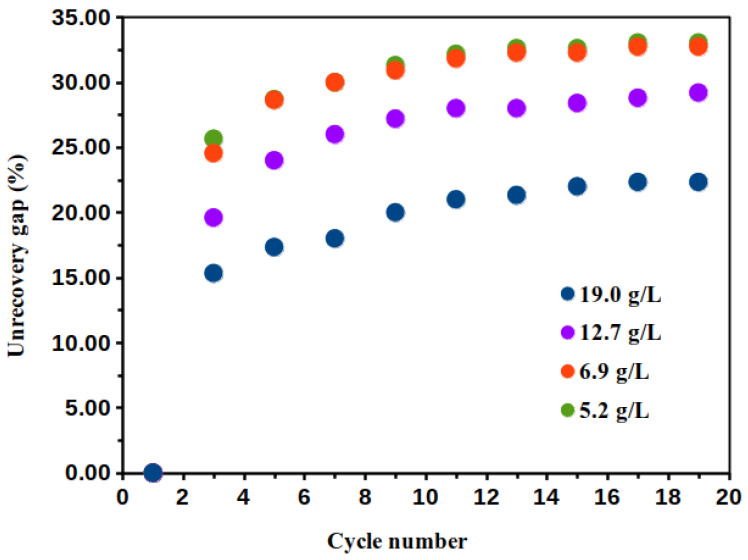
Unrecovered gap as a function of the odd compression cycles (cf. Figure 15) for the different effective concentration 5.2, 6.9, 12.7, and 19.0 g L^−1^ for APP-E at 1 mol L^−1^ CaCl_2_.

Figure 17 represents the « unrecovered gap » defined by: (h_0_ – h_n_)/h_0_, where h_0_ is the initial gap when the sample was first loaded and h_n_ is the zero-force gap at the nth cycle. The unrecovered gap converges to a plateau value, regardless of the concentration in pectin. It varies from 20 to 33 % in direct relation with the degree of swelling which increases when concentration decreases.

Finally, the pectin gel is surprising in that the slope at the small gap of the axial force as a function of the gap increases, leading to an E-modulus ~3 × 10^5^ Pa, twice as high as the E-modulus ~1.5 × 10^5^ Pa at the first cycle. The compression cycles induce a reinforcement of the gel which is correlated to a change in the 3-D structure.

In summary, compression allows: (i) to determine the elastic modulus for the first compression cycle in the nonlinear regime; (ii) to demonstrate an irreversible progressive plastic deformation of the gel; (iii) such a modulus to vary as a function of pectin concentration following E~C^1.75^, in accordance with the literature; (iiii) the elastic modulus to increase following a volume change, which stabilizes after a large number of compression/traction cycles (12 cycles). This evolution reflected a change in the gel morphology that was attributed to an increase in pectin chains associated with the presence of calcium [42].

## 4. Conclusions

In this work, the Araçá pectin was purified following a method previously selected with few modifications. Purified pectin was progressively demethylated and all the samples obtained were characterized by their DM (using titration and ^1^H-NMR) and M*_w_* distribution by SEC. It was confirmed that the basic conditions adopted for demethylation do not modify the molecular weight. The different parameters playing a role in gelation were investigated after dialysis of a given volume of pectin solution against CaCl_2_ using a dialysis cassette. The viscosity of ACP was not influenced by the presence of calcium, in accordance with its large DM (like a HM pectin), and APP and APP-A form heterogeneous systems based on large, gelled aggregates. Rheological behavior was confirmed by flow and dynamic experiments, and it was found that shear viscosity is lower than the dynamic viscosity. Considering the influence of DM throughout the range, a sharp transition of G′ and G″ of around DM < 30% was demonstrated, as found previously on different systems.

A prepared pectin concentration is important to obtain a gel. Firstly, the effective concentrations (C_eff_) in the gel were determined by a contraction of the systems, increasing with the initial polymer concentration. It is estimated that G′~C_eff_^2.8^ is in accordance with the coefficient found for other physical gels. The critical concentration C*, in order to obtain a homogeneous gel, was over 2 g L^−1^ (or C_eff_ > 5.2 g L^−1^). At the end, the influence of the CaCl_2_ concentration adopted for dialysis was studied: loose gels (large swelling degrees) were obtained against a large excess of 0.1 mol L^−1^ CaCl_2_; then, the degree of swelling decreased slowly when the ionic concentration increased up to 0.5 mol L^−1^. In this range of calcium concentration, the rheological behavior was the same. In 1 mol L^−1^ CaCl_2_, the swelling degree decreased strongly, and the effective concentration increased with a concomitant increase in G′ and G″ moduli. It is proposed that interchain long-range electrostatic repulsions counterbalance the calcium cross-linkage potential.

The traction/compression cycle test showed the behavior of a physical gel where an unrecovered gap occurs during the fatigue test. Moreover, the elastic modulus was in accordance with the literature and more likely follows a model of rigid polymer chains. The change of dissipation during the cycle is undoubtedly attributed to the rearrangement of pectin chain association inside the gel.

In conclusion, these results allow the discussion of the LM pectin behavior of an excess of calcium in the linear (viscoelastic characteristics) and nonlinear regimes (E-modulus in compression/traction).

## Figures and Tables

**Table 1:** Galacturonic acid and DM of pectins obtained from the Araçá pulp.

	Uronic Acid			
Sample	GalA-Na^+^ (×10^−3^) ^a^	GalA-Na^+ b^	GalA-Met ^b^	DM (%) ^a^
APP	184.6	36.5	50.5	59.0
APP-A *	281.8	55.8	31.2	36.8
APP-B *	298.4	58.7	28.3	33.0
APP-C *	453.1	89.7	0	0
APP-D *	439.4	87.0	0	0
APP-E *	429.0	85.0	0	0

^a^ Quantified by conductometric titration. GalA-Na^+^—expressed in number of -COO^−^ per 100 g of dried sample. DM: degree of methyl-esterification, expressed in molar ratio ×100. ^b^ GalA-Met, free Gal-A expressed in g/100 g of dried sample. * De-esterification reaction times: 15 min (APP-A); 30 min (APP-B); 45 min (APP-C); 60 min (APP-D); and 105 min (APP-E).

**Table 2:** Structural characterization of the different pectin samples by ^1^H-NMR.

Samples	DM ^a^	Acetyl ^a^	Rhamnose ^a^	Arabinogalactan ^b^	DM (%) Titration
APP	69.0	3.0	4.8	Nd	59.0
APP-A *	48.0	1.6	4.0	17.0	36.8
APP-B *	37.6	1.2	5.5	15.0	33.0
APP-C *	22.7	1.3	4.7	18.0	0
APP-D *	14.7	1.4	3.4	---	0
APP-E *	14.0	1.4	3.4	----	0

^a^ % mol/mol total GalA, obtained by ^1^H-NMR. ^b^ % Monosaccharide units/total GalA. * De-esterification reaction times: 15 min (APP-A); 30 min (APP-B); 45 min (APP-C); 60 min (APP-D); and 105 min (APP-E).

**Table 3:** SEC characterization of pectins after progressive demethylation dissolved in NaNO_3_ and water. M*_w_* is taken for all the chromatograms.

Samples	M*_w_* (g mol^−1^) in Salt	% Eluted Salt	M*_w_*(g mol^−1^) in Water	% Eluted Water
APP	1.94 × 10^6^	70.0	708,600	55.0
APP-A *	1.6 × 10^6^	61.3	978,000	68.1
APP-B *	698,000	75.8	561,400	71.9
APP-C *	680,000	81.0	506,300	78.7
APP-D *	------	--------	459,700	88.5
APP-E *	646,000	79.5	436,800	81.3

* De-esterification reaction times: 15 min (APP-A); 30 min (APP-B); 45 min (APP-C); 60 min (APP-D); and 105 min (APP-E).

**Table 4:** The role of DM on the effective polymer concentration and on the degree of gel swelling formed by dialysis of 10 g L^−1^ solutions against 1 mol L^−1^ CaCl_2_.

Samples	Initial Concentration (g L^−1^)	Effective Concentration (g L^−1^)	Degree of Swelling (mL g^−1^)
APP-B	10	20. 3	49.2
APP-E	10	19.0	52.6

**Table 5:** The role of APP-E pectin concentration on the effective polymer concentration and degree of swelling of gel formed by dialysis against 1 mol L^−1^ CaCl_2_.

Initial Concentration (g L^−1^)	Effective Concentration (g L^−1^)	Degree of Swelling (mL g^−1^)
2.0	5.2	192.3
3.3	6.9	146.3
6.7	12.7	78.7
10.0	19.0	52.6

**Table 6:** Effective polymer concentration and degree of swelling of gel formed from a 10 g L^−1^ solution of APP-E. Role of CaCl_2_ concentration.

CaCl_2_ Concentration (mol L^−1^)	Effective Concentration (g L^−1^)	Degree of Swelling (mL g^−1^)
0.10	11.7	85.7
0.25	11.7	83.2
0.50	13.6	73.1
1.00	19.0	52.6
